# Geroscience-Guided Repurposing of FDA-Approved Drugs to Target Aging: A 2025 Update

**DOI:** 10.1093/geroni/igaf122.1580

**Published:** 2025-12-31

**Authors:** George Kuchel

**Affiliations:** UConn Health, Farmington, Connecticut, United States

## Abstract

Common chronic diseases represent the greatest driver of rising healthcare costs, as well as declining function, independence, and quality of life. Geroscience-guided approaches seek to delay the onset and progression of multiple chronic conditions by targeting fundamental biological pathways of aging. This approach is more likely to improve overall health and function in old age than treating individual diseases, by addressing aging the largest and mostly ignored risk factor for the leading causes of morbidity in older adults. Nevertheless, challenges in repurposing existing and moving newly discovered interventions from the bench to clinical care have impeded the progress of this potentially transformational paradigm shift. In a 2002 Aging Cell article, we proposed the creation of a standardized process for evaluating FDA-approved medications for their geroscience potential. Criteria for systematically evaluating the existing literature that spans from animal models to human studies will permit the prioritization of efforts and financial investments for translating geroscience and allow immediate progress on the design of the next Targeting Aging with MEtformin (TAME)-like study involving such candidate gerotherapeutics. This talk will provide an update on scientific developments which have taken place over the last 3 years with a particular emphasis on potential “off-target” gerotherapeutic benefits of more recent diabetes drugs such as SGLT2 inhibitors and GLP1R agonists on conditions as varied as congestive heart failure, kidney disease, frailty, and vulnerability to severe COVID-19. Moreover, a novel Cochrane-like approach to evaluating the quality of varied observational and causal inference studies will also be discussed.

